# Biomechanical Comparison of Dorsal Wrist Spanning Plates Versus External Fixation in Distal Radius Fractures With a Simultaneous Axial and Bending Load

**DOI:** 10.1177/15589447251376587

**Published:** 2025-10-16

**Authors:** Umar Ghilzai, Aaron Singh, Jamie Alexander, Scott Mitchell, David TJ Netscher

**Affiliations:** 1 Department of Orthopedic Surgery, Baylor College of Medicine, Houston, TX, USA; 2 Department of Orthopedic Surgery, The University of Texas at Austin, USA; 3 Department of Orthopedic Surgery, Houston Methodist Hospital, TX, USA

**Keywords:** hand, fracture/dislocation, diagnosis, distal radius, biomechanics, basic science, wrist, trauma

## Abstract

**Introduction:**

External fixators have traditionally been used for comminuted distal radius fractures not amenable to plate fixation but are associated with complications. Dorsal spanning plates have emerged as an alternative. Prior biomechanical studies comparing these constructs typically assess forces in a single vector. This study compares dorsal spanning plates and external fixators under combined axial loading with simultaneous flexion, extension, radial, and ulnar bending to better replicate physiologic wrist forces.

**Methods:**

24 identical laser-sintered bone models were created, with 12 assigned to the dorsal spanning plate group and 12 to the external fixator group. Constructs were tested using a servohydraulic test machine under combined axial and bending loads. Stiffness, maximum load, and yield load were recorded. Statistical analysis was performed using the Mann-Whitney U test with Monte Carlo exact testing.

**Results:**

The dorsal spanning plate group demonstrated significantly greater stiffness than the external fixator group in radial and ulnar deviation. No significant differences in stiffness, maximum load, or yield load were observed between groups during flexion. In extension, the plate group exhibited significantly higher maximum load, while stiffness and yield load were not statistically different.

**Conclusion:**

Dorsal spanning plates demonstrate increased stiffness compared with external fixators under physiologic multivector loading. These findings may support their use in patients expected to load the wrist postoperatively, such as individuals with polytrauma or elderly patients reliant on assistive walking devices.

## Introduction

Distal radius fractures are among the most common fractures, representing approximately 10% of all extremity injuries and an incidence of more than 600 000 anually.^1,2^ These injuries have a bimodal distribution, occurring in younger patients sustaining high energy trauma, as well as older, osteoporotic patients from lower energy mechanisms.^3^ Higher energy injuries in particular may be associated with substantial soft tissue damage and fracture comminution.

Treatment of these injuries requires restoration of length, alignment, and congruency of the articular surface. Although volar plate fixation is now the most common modality used by surgeons in the United States, not all fractures are amenable to this construct. In particular, fractures offering limited fixation in the distal segment from poor bone stock, distal fracture lines, or extensive articular comminution may preclude stable plate fixation.^4^

Historically, external fixation has been used for these challenging fracture types. It aids in reduction via ligamentotaxis and maintains length until the fracture has healed or more definitive fixation can be achieved.^1,4^ However, external fixators are cumbersome for patients and associated with high complication rates including pin site infections, osteomyelitis, and injury of the superficial sensory branch of the radial nerve.^4^ More recently, dorsal wrist spanning plating systems have been described for patients with high energy fractures. The dorsal wrist spanning plate functions similarly to an external fixator, achieving reduction through ligamentotaxis, while mitigating many risks associated with external fixators.^1^ In addition, these plates can facilitate early load bearing in polytrauma patients or elderly patients who require assistive devices, facilitating ambulation and early functional use of the injured extremity.^5^

However, the amount of load that the dorsal spanning plate is able to withstand has not been well characterized in these situations and there is uncertainty regarding the safety of unrestricted weight-bearing. Several biomechanical studies comparing external fixator and dorsal wrist spanning plating have been performed. Chhabra et al^6^ found that a dorsal spanning plate was stiffer than external fixation with axial loading in acrylic rods. Wolf et al^7^ determined that a 2.4-mm spanning plate was more rigid than external fixation in a cadaver model with axial loading followed by flexion or extension loading through tendons. However, in vivo axial loading is seldom the only force acting across the wrist. Rather multiple forces, acting simultaneously, can result in loss of reduction.^8^ The goal of this study is to compare combined, simultaneous axial loading with flexion/extension bending moment to better simulate real-life forces across a wrist.

## Materials and Methods

### Bone Model Design

To establish a test model for the forearm, the Stryker Orthopedic Modeling and Analytics (SOMA) system, a proprietary bone database and software toolbox, was used.^9^ Forty-four upper extremity data set samples were chosen. These samples were from males and females between the ages of 19–95. Based on 3D-bone models, the bone lengths and volumes were calculated to obtain an average for the second and third metacarpals as well as radius. The specific specimen of these 44 data sets that most closely fit the bone averages was used as the representative template to generate the bone models.

**Figure 1.**
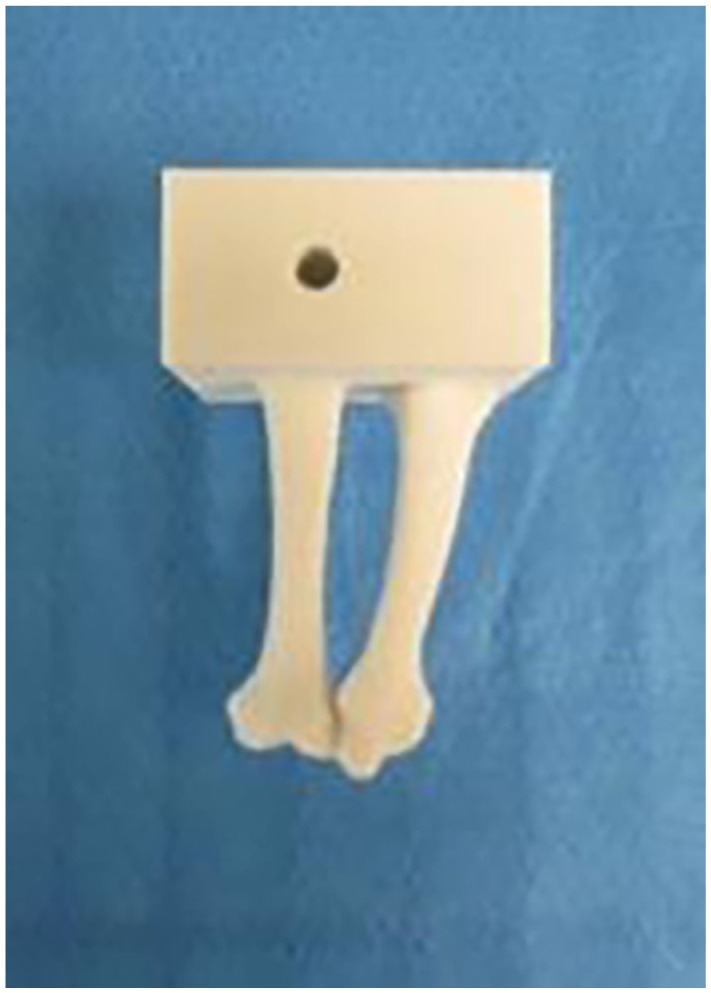
This is an anterior view of the metacarpal component of the laser sinter bone model.

**Figure 2.**
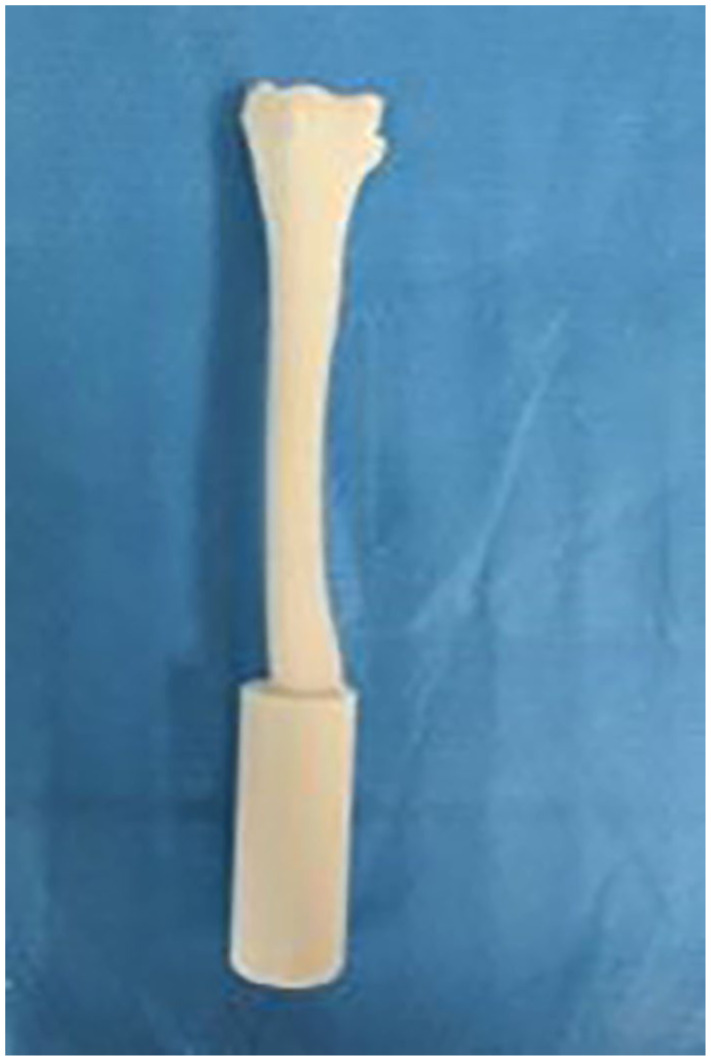
This is an anterior view of the radial component of the laser sinter bone model.

Once the bone model template was completed, the second and third metacarpals were selected and the remaining bones in the hand and wrist were digitally subtracted. The ulna was also digitally subtracted yielding a digital construct with 2 components, a radius component and a second and third metacarpal component. The 3D models were generated using Nylon 12 GF, a glass filled nylon material that is light weight but has stiffness similar to bone. The models were printed using a laser sintering process in which a laser is used to bind a powdered substrate based on a computer model to make a solid structure. This process resulted in 24 identical 2 component bone models (Figures 1 and 2) with anatomically compliant geometry.

### Sample Groups

The samples were divided into 4 groups with 6 samples in each group. Twelve samples were in the wrist spanning plating group with 2 subgroups: flexion and extension. Similarly, 12 samples were in the external fixator group with again 2 subgroups: 6 in the flexion group and 6 in the extension group.

### Assembly of the Bone Models

A jig was created to ensure anatomic alignment and spacing of the bone models. The bone model jig was secured to the work bench using C clamps (Supplement 1). The wrist spanning plates, the Stryker VariAx 2 Wrist Spanning Plate, and the Hoffman II Compact MRI External Fixator Systems were applied to the bone models while in the jig (Supplement 1).

The wrist spanning plate was applied on the dorsal-radial surface of the radius. This was fixated with 2.7-mm titanium screws. The initial screw was placed in the oblong hole with a cortical screw followed with 2 locking 2.7-mm titanium screws directly proximal and distal to the cortical screw. The distal portion of the plate was similarly fixated to the index metacarpal with 2.4-mm screws. A single cortical screw was placed in the oblong hole followed by 2-locking screws in the adjacent proximal and distal screw holes.

The Stryker Hoffman II Compact MRI External Fixator System was applied to the bone models in the jig template using 3-mm self-drilling pins. The radial shaft pins were placed 5- and 7-cm proximal to the proximal edge of Lister’s tubercle along the dorsal-radial portion of the shaft. The second metacarpal shaft pins were placed in the index metacarpal 2- and 4-cm distal to the proximal radial edge of the base of the metacarpal. A multipin clamp was then used to secure each of the 2 sets of pins. Thirty-degree posts were placed on both sides of this clamp followed by rod-to-rod couplings. Finally, a 5-mm rod was placed along the 2 sides of the fixator device. There was reuse of the multipin clamp, 30° posts, rod-to-rod couplings, and proximal self-drilling pins due to limitations in the number of implants available.

### Experimental Design

**Figure 3.**
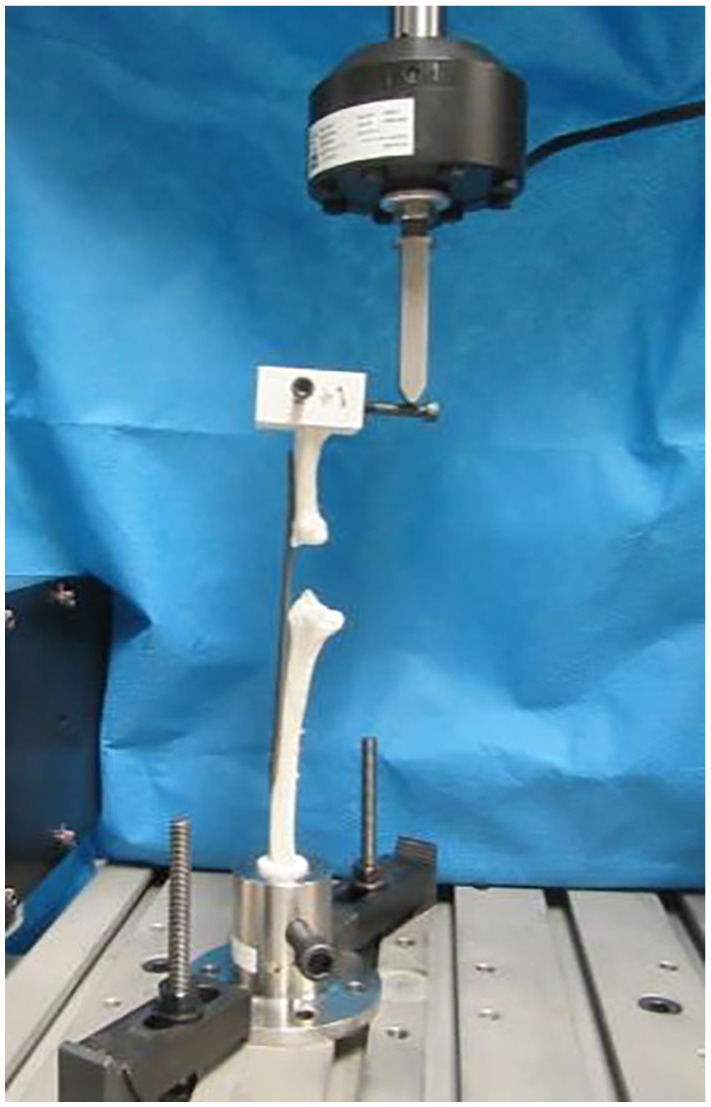
The bone models were secured in the servohydraulic test machine (*MTS Systems Corporation* – Eden Prarie, Minnesota) and the load was applied to the pin 55 mm to the central axis of the radius. This allowed for a simultaneous axial load and bending moment.

The completed models were placed in a servohydraulic test machine (*MTS Systems Corporation* – Eden Prarie, Minnesota) with a flexion or extension load placed 55 mm from the central axis of the radius (Figure 3). They were secured in place using clamps. The position of the load created a simultaneous axial compression and bending moment. The loading was applied at a rate of 3.5 mm/min. The testing for the sample was considered complete when there was ultimate failure of the implant or when there was a total of 50 mm of displacement (Supplement 2). The mode of failure, force, and displacement were recorded for each bone model. Each model was also tested under isolated radial and ulnar bending moments to measure directional stiffness independent of axial load.

**Figure 4.**
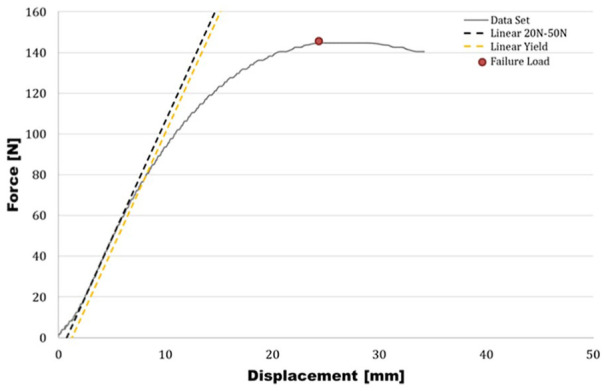
Force-displacement curve. *Note.* The maximum load was determined as the load at which catastrophic failure (breakage of bone or hardware) occurred or the apex of the force-displacement curve. Yield load was defined as the force at which the linear portion of the force-displacement curve transitioned from elastic (linear) to plastic deformation (curved).

All data points were recorded electronically and exported to Microsoft Excel (Microsoft, Redmond, WA). Stiffness, maximum load, and yield load were calculated. Stiffness was calculated from the raw data by determining the slope of the linear aspect of the force-displacement curve between 20 and 50 N (Figure 4). The maximum load was defined as the load at which catastrophic failure (breakage of bone model or hardware) occurred or the apex of the force-displacement curve (Figure 4). Yield load was defined as the force at which the construct showed a residual deformation of 0.5 mm (Figure 4).

### Statistical Analysis

Statistical analysis of the biomechanical test was performed using the Mann-Whitney U test including the Monte Carlo-exact test. The P-value was calculated using nonparametric statistics of the Monte Carlo estimate type on 10 000 samples with a 95% confidence interval. Significance level for all tests was 95% (alpha = .05)

## Results

### Flexion

**Figure 5.**
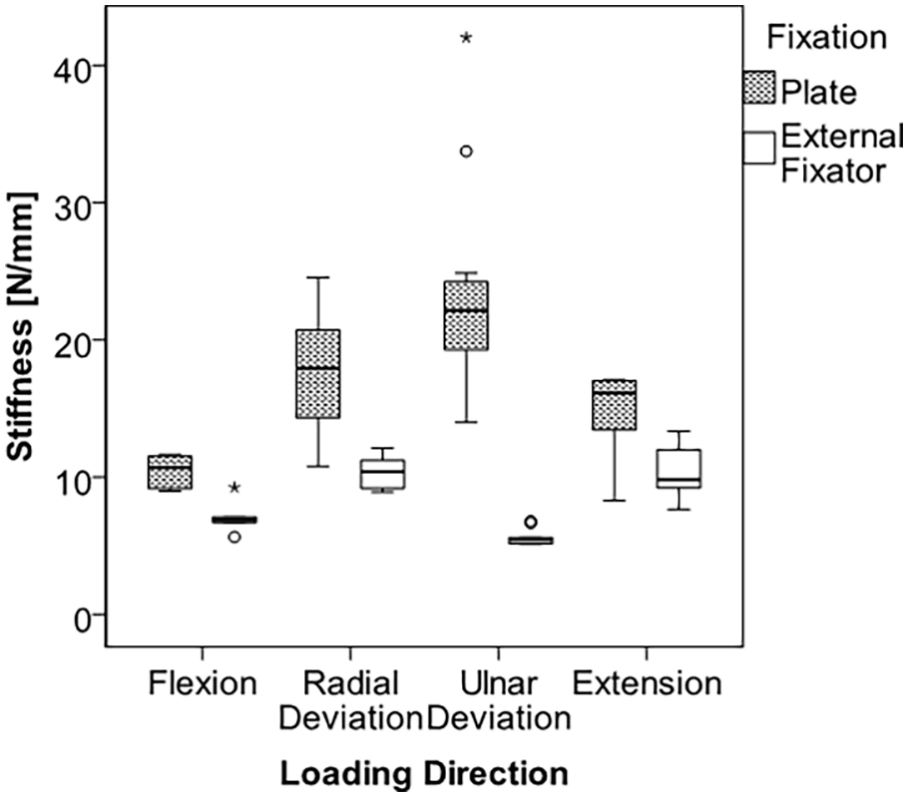
Box-and-whisker plot. *Note.* The box shows the interquartile range (IQR) and the whiskers extend to the smallest and largest values except data points with distance greater than 1.5 times IQR. The black line within the box represents the Median. Outliers are data points lying between 1.5 times IQR and 3 times IQR and were marked as circles (o). Extremes are data points beyond 3 times IQR and were marked as asterisks (*). All data points were included for statistical analysis.

There was no statistical difference in maximum load, maximum bending moment, yield load, or yield bending moment in flexion between external fixators and wrist spanning plates (Table 1). There was no significant difference in stiffness between the 2 implant types (*P* = .08) (Table 2). The average stiffness of the spanning plates was 10.46 N/mm, and the median value of 10.71 N/mm with a standard deviation of 1.15 N/mm compared with an average of 7.08 N/mm with a median value of 6.88 N/mm and a standard deviation of 1.18 N/mm for the external fixators (Figure 5).

**Table 1.** Summary Table of Maximum and Yield Load in Flexion and Extension for Plate and External Fixator Groups.

**Motion**	**Construct**	**Sample size**	**Maximum load (N) ± *SD***	***P*-value (maximum load)**	**Yield load (N) ± *SD***	***P*-value (yield load)**
**Flexion**	Plate	6	135.91 ± 7.77	.93	64.99 ± 4.16	.94
	External Fixator	6	127.07 ± 23.40		63.03 ± 11.03	
**Extension**	Plate	6	154.10 ± 5.92	<.01	75.03 ± 2.52	.72
	External Fixator	6	120.84 ± 14.03		72.52 ± 6.24	

**Table 2.** Summary Table of Construct Stiffness in Flexion, Extension, Radial, and Ulnar Deviation for Plate and External Fixator Groups.

**Motion**	**Construct**	**Sample size**	**Average stiffness (N/mm) ± *SD***	**Median (interquartile range)**	***P*-value**
Flexion	Plate	6	10.46 ± 1.15	10.71 (2.43)	.08
	External Fixator	6	7.08 ± 1.18	6.88 (1.21)	
Radial Deviation	Plate	12	17.94 ± 4.13	−	<.01
	External Fixator	10	10.34 ± 1.05	−	
Ulnar Deviation	Plate	12	23.69 ± 7.48	−	<.01
	External Fixator	9	5.67 ± 0.64	−	
Extension	Plate	6	14.70 ± 3.43	16.14 (10.31)	.42
	External Fixator	6	10.31 ± 2.04	9.83 (3.50)	

### Radial/Ulnar Deviation

Significant differences were observed in stiffness in radial and ulnar deviation between the plating and external fixator cohorts. The stiffness in radial deviation for plates averaged 17.94 N/mm versus the external fixators which was 10.34 N/mm (*P* < .01). In ulnar deviation, the stiffness of the plates was greater than the external fixators with values of 23.69 and 5.67 N/mm, respectively (*P* < .01) (Table 2).

### Extension

In extension, there was no statistically significant difference in stiffness (*P* = .42) (Table 2). Plates had an average stiffness of 14.70 N/mm with a standard deviation of 3.43 N/mm compared with 10.31 N/mm with a standard deviation of 2.04 N/mm for the external fixators. Maximum load and maximum bending moment were both greater in the spanning plates compared external fixators, with values of 154.10 versus 120.84 N (*P* < .01) for maximum load and 8.48 versus 6.65 Nm (*P* < .01) for maximum bending moment (Table 1). No statistically significant difference could be found with regard to yield load and yield bending moment.

## Discussion

External fixators have traditionally been used for severely comminuted distal radius fractures not amenable to open reduction internal fixation with standardized plating.^10^ However, pin site complications including loosening, pin site infection, osteomyelitis, digital stiffness, and patient dissatisfaction limit the appeal of external fixation.^11,12^ Also, an external fixator’s decreased rigidity may prolong healing time in distal radius fractures.^13^ These combined factors can lead to inadequate structural support in the timeline needed for a distal radius fracture to heal without displacement. Also, it has been shown that wrist spanning plates may be more stable and have greater stiffness compared with external fixators.^4,7^

Prior studies have focused on axial loading or axial loading followed by the various bending forces.^6,7^ However, these studies do not fully capture the typical simultaneous forces across a wrist. This study aimed to simulate a more lifelike scenario of simultaneous bending and axial loading. In addition, this model removes all other variables that could possibly offer support across the wrist (carpus, skin, tendons, ligaments, etc) to accurately compare the constructs (external fixator and spanning plate).

Under these conditions, the spanning plate demonstrated greater stiffness when simultaneously loaded axially in combination with radial and ulnar deviation, and trended toward greater stiffness in flexion and extension when compared with the external fixator. Notably, the dorsal spanning plate demonstrated significantly greater load-bearing capacity—reflected by higher maximum load and bending moment—in extension, a position relevant to tasks such as ambulating with a walker or using assistive devices. Still, both constructs were able to withstand forces well above 100 N across the wrist, which is consistent with forces generated during light active range of motion.^12^ In relation to flexion/extension and radioulnar movements, Winemaker et al^14^ found that these bending moments during most daily activities remained below 3.5 Nm, indicating both constructs are likely to support the wrist during light routine activity. These findings may help surgeons feel more confident recommending early functional use and limited weight bearing, and improve postoperative counseling.

This study has limitations. This study was performed using synthetic bone material. Although the bone model approximates the qualities of real bone, it was manufactured based on the characteristics of 44 distinct bone models for the wrist and may not fully reflect the variation in bone quality of the population. In addition, this model was not set up to test for differences that may occur in osteoporotic bone which is present in a significant proportion of distal radius fractures. However, for the purposes of this study, it was necessary to minimize the impact of differing bone quality, which may influence the behavior of the bone-implant interface. Furthermore, many of the biomechanical properties of the implants tested are intrinsic to the construct, regardless of bone quality; therefore, this study may still provide valuable data to surgeons treating highly heterogeneous populations.

The lack of a soft tissue envelope, which was a variable that was intentionally excluded from this study, may reduce the absolute number for yield load and maximum load and stiffness as the soft tissue can add additional support. In addition, because the tendons were not included in this model they could not be used as a stabilizing or antagonistic force to extend the model’s applicability to in vivo situations. Also, the external fixator configuration was more robust than the typical 1 bar/4 pin technique frequently used in clinical practice. In this model, a 2-bar configuration was used per manufacturers instruction for use that offers a more stable construct than the 1 bar/4 pin technique.

## Conclusion

This study further supports dorsal wrist spanning plates for distal radius fractures due to the increased stiffness of the plates when loading with simultaneous axial load and bending moment when compared with the external fixator. Since these forces more closely simulate the forces across the wrist, this may allow for more confidence in using spanning plates in patients that will need to exert load across the wrist. Although this study focused on comparing the rigidity and strength (static) of the 2 constructs, future studies may be focused on using cyclic loading in a cadaveric model to more closely approximate physiologic loading.^15^

## Supplemental Material

sj-docx-1-han-10.1177_15589447251376587 – Supplemental material for Biomechanical Comparison of Dorsal Wrist Spanning Plates Versus External Fixation in Distal Radius Fractures With a Simultaneous Axial and Bending Load Supplemental material, sj-docx-1-han-10.1177_15589447251376587 for Biomechanical Comparison of Dorsal Wrist Spanning Plates Versus External Fixation in Distal Radius Fractures With a Simultaneous Axial and Bending Load by Umar Ghilzai, Aaron Singh, Jamie Alexander, Scott Mitchell and David TJ Netscher in HAND

sj-docx-2-han-10.1177_15589447251376587 – Supplemental material for Biomechanical Comparison of Dorsal Wrist Spanning Plates Versus External Fixation in Distal Radius Fractures With a Simultaneous Axial and Bending Load Supplemental material, sj-docx-2-han-10.1177_15589447251376587 for Biomechanical Comparison of Dorsal Wrist Spanning Plates Versus External Fixation in Distal Radius Fractures With a Simultaneous Axial and Bending Load by Umar Ghilzai, Aaron Singh, Jamie Alexander, Scott Mitchell and David TJ Netscher in HAND
