# Supplementary material for: Biomechanical Comparison of Dorsal Wrist Spanning Plates Versus External Fixation in Distal Radius Fractures With a Simultaneous Axial and Bending Load
Source: Hand (N Y). 2025 Oct 16;21(8):1280–5. doi: 10.1177/15589447251376587 (PMC12534833; doi:10.1177/15589447251376587)
Supplement: Supplementary file 1 [file sj-docx-1-han-10.1177_15589447251376587.docx]

Supplement 1: The spanning plates and external fixators were applied to the bone models in the jig.
