# Supplementary material for: Biomechanical Comparison of Dorsal Wrist Spanning Plates Versus External Fixation in Distal Radius Fractures With a Simultaneous Axial and Bending Load
Source: Hand (N Y). 2025 Oct 16;21(8):1280–5. doi: 10.1177/15589447251376587 (PMC12534833; doi:10.1177/15589447251376587)
Supplement: Supplementary file 2 [file sj-docx-2-han-10.1177_15589447251376587.docx]

Supplement 2: The load was applied at a rate of 3.5 mm/min (Figure 4). The testing for the sample was considered complete when there was failure of the implant or when 50 mm of displacement was reached.
