# Supplementary material for: Biomechanical Comparison of Dorsal Wrist Spanning Plates Versus External Fixation in Distal Radius Fractures With a Simultaneous Axial and Bending Load
Source: Hand (N Y). 2025 Oct 16:15589447251376587. Online ahead of print. doi: 10.1177/15589447251376587 (PMC12534833; doi:10.1177/15589447251376587)
Supplement: sj-docx-1-han-10.1177_15589447251376587 – Supplemental material for Biomechanical Comparison of Dorsal Wrist Spanning Plates Versus External Fixation in Distal Radius Fractures With a Simultaneous Axial and Bending Load [file sj-docx-1-han-10.1177_15589447251376587.docx]

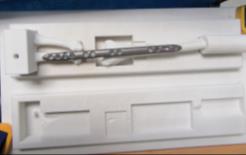

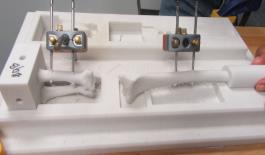


Supplement 1: The spanning plates and external fixators were applied to the bone models in the jig.
